# A delicate calculation method for reservoir initial water saturation based on a novel approach to J-function construction

**DOI:** 10.1371/journal.pone.0328726

**Published:** 2025-09-04

**Authors:** Xiongzhi Liu, Hao Yang, Yongqiang Qu, Liang Hong, Lifei Dong, Hao Kang

**Affiliations:** 1 China University of Geosciences (Beijing), Beijing, China; 2 Research Institute of Petroleum Exploration & Development-Northwest PetroChina, Lanzhou, China; 3 Chongqing Three Gorges University, Chongqing, China; 4 Polytechnic Institute, Hebei Normal University, Shijiazhuang, China; 5 Hebei Provincial Key Laboratory of Information Fusion and Intelligent Control, Shijiazhuang, China; Vietnam Maritime University, VIET NAM

## Abstract

Accurately determining initial water saturation is essential for assessing reservoir resources and optimizing development, yet conventional J-function methods face challenges such as variability in curve fitting due to sample heterogeneity, less-than-optimal grouping schemes, and limited core sample availability. This study addressed these issues by analyzing 55 J-function curves from the N reservoir in Iraq’s Zagros Basin, identifying permeability as the primary factor influencing curve shape. A novel classification method was developed, categorizing curves into power function and exponential function types based on permeability thresholds. Improved J-function models were established by correlating undetermined coefficients with the reservoir quality index (RQI), enabling continuous, tailored calculations, eliminating the need for sample grouping. Case studies demonstrated that the improved J-function method provides more accurate water saturation (S_w_) estimates in Reservoir N (porosity 0.1–0.27) compared to the Archie equation and conventional J-function: (1) In high-quality intervals (e.g., 3682–3683 m), it yields S_w_ = 0.06–0.11, closely matching core-derived irreducible water saturation (0.1), while Archie overestimates S_w_ (0.23–0.74); (2) In poor-quality zones (3676.5–3681 m), it agrees with Archie’s S_w_ but corrects the conventional J-function’s underestimation by accounting for subtle property variations. These advancements provided a robust framework integrating permeability-based J-function classification, RQI-correlated coefficient models, and continuous saturation calculations, eliminating the need for sample grouping.

## 1. Introduction

In the coming period, the global oil and gas market will continue to be in a complex environment full of changes and uncertainties [1–3]. Against this background, the efficiency of oil and gas resource exploration and development urgently needs to be further improved.

From a technical perspective, in the process of oil reservoir development, accurately determining the water saturation of the reservoir is crucial for assessing reservoir resources, optimizing development plans, predicting reservoir performance, and ensuring economic benefits. Specifically, in terms of reservoir resource assessment, by precisely measuring water saturation, the reserves of movable oil in the reservoir can be calculated more accurately, thereby evaluating the resource potential of the reservoir and providing a solid data foundation for oilfield development planning [4–6]. Development plans can be optimized based on reservoir water saturation, enabling tailored extraction methods and production strategies [7–9]. Moreover, in terms of predicting reservoir performance, accurately determining water saturation and combining it with actual data from the production process can better predict the changing trends of the oil-water interface during exploitation and the patterns of increasing water cut in the reservoir, thus providing a scientific basis for the dynamic monitoring and management of reservoir production [10–12].

Initial fluid saturation is a cornerstone of reservoir characterization, directly influencing reserves estimation, development strategy design, and performance prediction. Generally, the initial water saturation of the reservoir can be determined by geophysical logging methods and direct measurement after sealed coring [13–15]. However, these methods are somewhat limited in terms of cost and applicability. In experimental reservoir engineering methods, the J-function has the advantages of wide applicability and high reliability of calculation results in determining the initial water saturation of the reservoir. Despite limitations such as sensitivity to core heterogeneity, the need for accurate grouping, and reliance on sufficient core data, it is advantageous for normalizing capillary pressure data across different rock types thus a relatively effective method and has important application in the field of oil exploration and development.

The J-function has been a cornerstone in reservoir engineering and petrophysics for decades, providing a robust framework for normalizing capillary pressure data and improving reservoir characterization. J-function is put forward by M. C. LEVERETT in year 1941 [16]:


$$J\left(S_{\text{w}}\right)=\frac{P_{\text{c}}}{\sigma \;\text{cos}\;\theta }\sqrt{\frac{K}{\emptyset }}$$ (1)

In the formula, $S_{\text{w}}$—Water saturation; $P_{\text{c}}$—Measured capillary pressure, MPa; σ—Interfacial tension, mN/m; θ—Wetting angle, °; *K*—Permeability, 10^−3^μm^2^ as the SI unit (mD as the field unit and 1mD ≈ 0.987 × 10^−3^μm^2^); ∅—Porosity; $\sqrt{\frac{K}{\emptyset }}$—Reservoir quality index (RQI), μm。

The J-function curve is a “scatter curve” composed of water saturation and J-function in a rectangular coordinate system. At present, the method of qualitative description is mainly used to study the morphological characteristics and influencing factors of capillary force curve or J-function curve. The shape characteristics of J-function curve are mainly controlled by the sorting of pore throat and the size of throat. The more concentrated the throat size distribution is, the better the sorting is, and the longer the middle flat segment of the capillary force curve is, and the closer it is to parallel to the abscissa axis. The size and concentration of the pore throat mainly affect the skewness of the curve, which is a measure of whether the shape of the capillary pressure curve deviates from the thick throat or the thin throat. The larger the throat is and the more the large throat is, the closer the curve is to the lower left of the coordinate plane, which is called coarse skew. On the contrary, the curve is closer to the upper right of the coordinate plane, which is called fine skew [17–19]. The shape of J-function curve mainly appears as power function type, exponential function type, and so on [20–22].

The method of calculating reservoir water saturation by using J-function has been widely used [22–25].

For power function type J-function, the principle of calculating water saturation is as follows:


$$J\left(S_{\text{wn}}\right)=\frac{mP_{\text{clab}}}{\sigma_{\text{lab}}\;\text{cos}\;\theta_{\text{lab}}}\sqrt{\frac{K}{\emptyset }}$$ (2)

$$J\left(S_{\text{wn}}\right)=a{S_{\text{wn}}}^{\text{b}}$$ (3)

From formula (2) and formula (3):


$$\frac{mP_{\text{clab}}}{\sigma_{\text{lab}}\;\text{cos}\;\theta_{\text{lab}}}\sqrt{\frac{K}{\emptyset }}=a{S_{\text{wn}}}^{\text{b}}$$ (4)

Capillary force, interfacial tension and contact angle under experimental conditions and reservoir conditions have the following relationship:


$$\frac{P_{\text{clab}}}{\sigma_{\text{lab}}\;\text{cos}\;\theta_{\text{lab}}}=\frac{P_{\text{cres}}}{\sigma_{\text{res}}\;\text{cos}\;\theta_{\text{res}}}$$ (5)

Substitute Formula (5) into Formula (4) to obtain:


$${S_{\text{wn}}}^{b}=\frac{mP_{\text{cres}}}{\sigma_{\text{res}}\;\text{cos}\;\theta_{\text{res}}}\sqrt{\frac{K}{\emptyset }}$$ (6)

The relationship between the capillary force and the oil column height is as follows:


$$P_{\text{cres}}=n\Delta \rho g\Delta H$$ (7)

Substitute Formula (7) into Formula (6) to obtain:


$$S_{\text{wn}}={\left(\frac{mn\Delta \rho g\Delta H}{a\sigma_{\text{res}}\;\text{cos}\;\theta_{\text{res}}}\sqrt{\frac{K}{\emptyset }}\right)}^{\frac{\text{1}}{\text{b}}}$$ (8)

The normalized irreducible water saturation is given by:


$$S_{\text{wn}}=\frac{S_{\text{w}}-S_{\text{wi}}}{1-S_{\text{wi}}}$$ (9)

Substitute Formula (9) into Formula (8) to obtain:


$$S_{\text{w}}=S_{\text{wi}}+(1-S_{\text{wi}}){\left(\frac{mn\Delta \rho gH}{a\sigma_{\text{res}}\;\text{cos}\;\theta_{\text{res}}}\sqrt{\frac{K}{\emptyset }}\right)}^{\frac{\text{1}}{\text{b}}}$$ (10)

Exponential function type J-function is shown in formula (11):


$$J\left(S_{\text{wn}}\right)=ce^{\text{d}S_{w}}$$ (11)

Similarly, the model for calculating the corresponding initial water saturation of the reservoir is shown in Formula (12):


$$S_{\text{w}}=S_{\text{wi}}+\left(1-S_{\text{wi}}\right)\frac{\text{ln}(\frac{mn\Delta \rho gH\;}{{c\sigma }_{\text{res}}\;\text{cos}\;\theta_{\text{res}}\;}\sqrt{\frac{K}{\emptyset }})}{d}$$ (12)

Where: *J*— Leverett J-function, dimensionless; $P_{\text{clab}}$— measured capillary force under laboratory conditions, psi; $\sigma_{\text{lab}}$— interfacial tension between mercury and water under laboratory conditions, dyn/cm; $\theta_{\text{lab}}$— contact angle between mercury and air under laboratory conditions; °; $P_{\text{cres}}$— oil-water capillary force under reservoir conditions, psi; $\sigma_{\text{res}}$— interfacial tension between oil and water under reservoir conditions, dyn/cm;$\theta_{\text{res}}$— contact angle between oil and water under reservoir conditions, °; Δρ— density difference between oil and water, g/cm^3^; ΔH— height of oil column, m; *g* — acceleration of gravity, m/s^2^; *m*— conversion coefficient between SI and field units for capillary pressure, 0.2166; *n*— conversion coefficient between SI and field units for fluid density, 1.4214; *a*—the undetermined coefficient of the power function type J-function; *b*—the undetermined coefficient of the power function type J-function; *c*— the undetermined coefficient of the exponential function type J-function; *d*— the undetermined coefficient of the exponential function type J-function.

Generally, accurate water saturation (S_w_) determination is hindered by two key limitations: (1) core data scarcity for J-function construction, and (2) errors introduced by subjective curve grouping—issues this study directly addresses. Peng et al. [26] improved the J-function model at a theoretical level. The authors derived the theoretical expression of the J-function for the first time using a rock capillary bundle model, overcoming the limitations of traditional semi-empirical formulas. They proposed that the J-function fitting should adopt a power function form and verified this through case studies. Additionally, they derived a theoretical expression for relative permeability calculations based on the J-function, providing new directions for its application. Xue et al. [27] developed a numerical model for average capillary pressure saturation using the J-function to address challenges in low-permeability reservoir development. Their findings showed that capillary pressure, as a driving force in water-wet reservoirs, promotes more uniform vertical distribution of oil and gas, with weakly water-wet rocks exhibiting the best waterflooding efficiency. The model quantitatively revealed the mechanism of capillary pressure effects on displacement by eliminating the influence of reservoir properties. Sima et al. [28] tackled the challenge of saturation calculation in heterogeneous carbonate reservoirs. Using data from a Middle Eastern oilfield, including capillary pressure curves, petrophysical data, and RFT test results, they established a J-function-based saturation calculation method. This approach yielded saturation values consistent with relative permeability experiments and production tests, even in the absence of core saturation data. Dai et al. [29] innovatively proposed a direct J-function fitting method for offshore oilfields. By integrating capillary pressure data from multiple cores to mitigate heterogeneity effects and combining it with a porosity model, they constructed a 3D saturation field. This method overcame the limitations of sparse well data, providing a reliable fluid distribution model for optimizing offshore field development plans. Yang [30] established a 3D oil saturation model based on the J-function to meet the needs of waterflooded oilfield development. By converting capillary pressure curves into J-functions and incorporating parameters such as oil column height, the model more accurately reflected the original oil-bearing state, supporting reserve reassessment and reservoir simulation. Shao et al. [31] enhanced the accuracy of saturation evaluation in tight sandstone gas reservoirs. By classifying capillary pressure curve shapes and establishing statistical relationships between J-functions and saturation, their new method doubled the accuracy compared to the traditional Archie equation. It particularly improved the characterization of saturation transitions near gas-water contacts, offering a practical solution for saturation determination in low-porosity, low-permeability reservoirs. Gonzalez et al. [32] developed a water saturation model to improve hydrocarbon reserve estimates by incorporating initial water saturation (S_wi_) in transition zones, traditionally overlooked when using irreducible water saturation (S_wirr_) in OOIP calculations. Leveraging the Leverett J-function—which integrates rock/fluid properties (e.g., permeability, porosity, fluid density)—the team derived S_WINIT_ = f(J) relationships from core capillary pressure data. Validated by production tests, the model quantified S_wi_ using original oil-water contact and rock quality, reducing OOIP overestimation by ~5% compared to S_wirr_-based methods. It also enabled reservoir simulation initialization (“Reservoir A”) independent of well-drilling timelines, enhancing accuracy in fluid distribution modeling. Ferreira et al. [33] developed an advanced pore-throat characterization method for complex carbonate reservoirs. Their key innovations include: (1) A truncated multi-Gaussian decomposition of pore-throat distributions, validated using global MICP data, which better captures multimodal heterogeneity than Thomeer’s method; (2) A novel multi-Gaussian universal J-function that reconstructs capillary pressure curves; and (3) A dynamic rock-typing index. Applied to Brazilian Pre-Salt analogs, the method demonstrated that pore-throat multimodality significantly impacts IOR/EOR recovery predictions (5–15% variance in OOIP estimates). The technique improves simulation accuracy by incorporating pore-network geometry effects often overlooked in conventional analyses.

Although the above method of using J-function to calculate the water saturation of reservoir has been widely used, there are still some shortcomings in the application effect. Firstly, the physical properties of cores tested by mercury injection are often quite different, which leads to the dispersion of J-function curves and makes it difficult to fit J-function curves in the following steps. In order to improve the fitting accuracy of the J-function curve, the J-function curve is usually divided into groups based on porosity, permeability, reservoir quality index, flow unit index and R_35_ [20,24,25,34]. This grouping method does not appropriately classify the J-function curves in advance, and it is very likely that different types of J-function curves are divided into the same group. In fact, only the same type of J-function curve can be divided into the same group. Second, the grouping scheme of the conventional J-function curve is difficult to achieve the optimum results. If there are too few groups, the number of J-function curves contained in each group is too large, and the J-function curves are not concentrated enough, resulting in low fitting accuracy of the J-function curve; if there are too many groups, the number of J-function curves contained in each group is too small, and the uncertainty is increased. Moreover, the J-function curve after grouping is still not enough to reflect the heterogeneity of the reservoir. Thirdly, due to limited coring, the J-function curves corresponding to some reservoirs cannot be obtained. Fourth, because the core is too loose, when it is taken to the surface, it has become loose sand, and the mercury injection experiment can not be carried out, resulting in the failure to obtain its J-function curve, and then the J-function curve of this kind of high-quality reservoir can not be obtained. This phenomenon exists in N reservoir of Zagros Basin in Iraq. Fifth, for the J-function curves of the same reservoir, even if they are grouped, only a single mathematical model [20,22] is used (for example, only the power function model or only the exponential function model is used), resulting in low fitting accuracy.

In order to solve the above problems, firstly, the shape characteristics and influencing factors of J-function curves are studied qualitatively, and the classification method of J-function curves is established. Then, by improving the undetermined coefficients of the conventional J-function, an improved J-function and a new method for calculating the initial water saturation of the reservoir are established.

## 2. Methodology

### Characteristics, influencing factors and classification of J-function curve

#### 2.1.1. Characteristics analysis of J-function curve.

Experimental cores were from the N sandstone reservoir of the Zagros Basin in Iraq, totaling 55 pieces. The porosity distribution ranges from 0.07 to 0.23, with an average of 0.18. The permeability ranges from 2.01 × 10^−3^μm^2^ to 2604.54 × 10^−3^μm^2^, with an average of 658.05 × 10^−3^μm^2^. Mercury injection data utilized for J-function calculation was supplied by Core Lab, USA, ensuring standardization and reliability. The termination pressure for mercury injection is 55000 psi, and at the end of mercury injection, water saturation was almost zero, so S_wn_ in Equation (9) is approximately equal to S_wi_.

**Fig 1:**
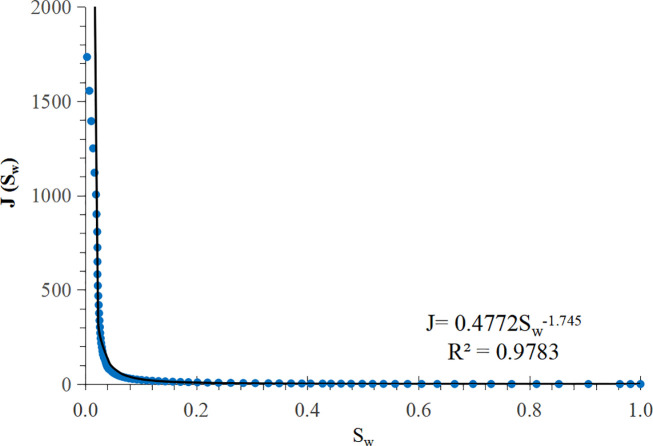
A power function type J-function curve.

**Fig 2:**
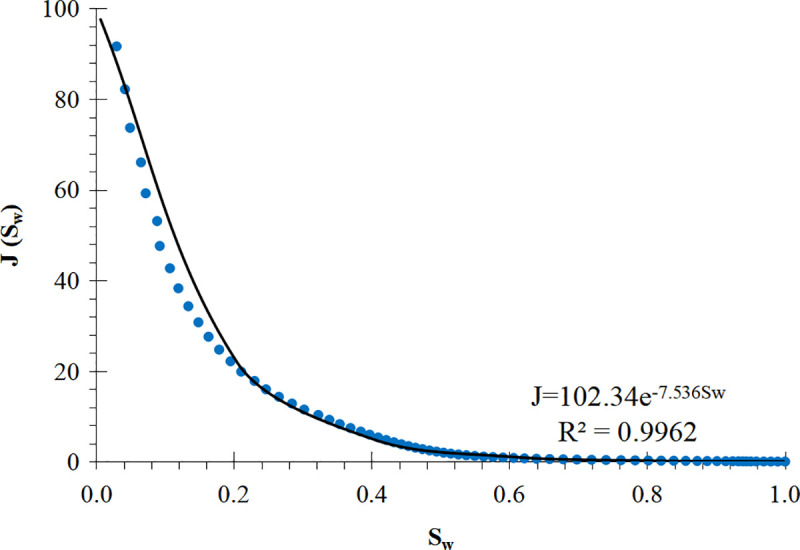
An exponential function type J-function curve.

The least square method is used to fit the J-function curve of each core mentioned above to obtain a corresponding mathematical model. According to this mathematical model, a curve called “pseudo J-function curve” can be generated. Typical pseudo J-function curves are shown in Figs 1 and 2. Fig 1 shows the J-function curve of the power function type, which has a long straight section, extending from right to left, almost to the vicinity of the irreducible water saturation, and suddenly becomes steep when approaching the irreducible water saturation. Fig 2 shows the J-function curve of the exponential function type, which has almost no straight section, or the straight section is very short and rises slowly from right to left. In this study, the pseudo-J-function curves of 55 cores were generated by using the above method, and then the curve types were classified based on their shapes: 35 cores’ J-function curves were power function type, and 20 cores’ J-function curves are exponential function type.

#### 2.1.2. Influencing factors and classification of J-function curve.

**Fig 3:**
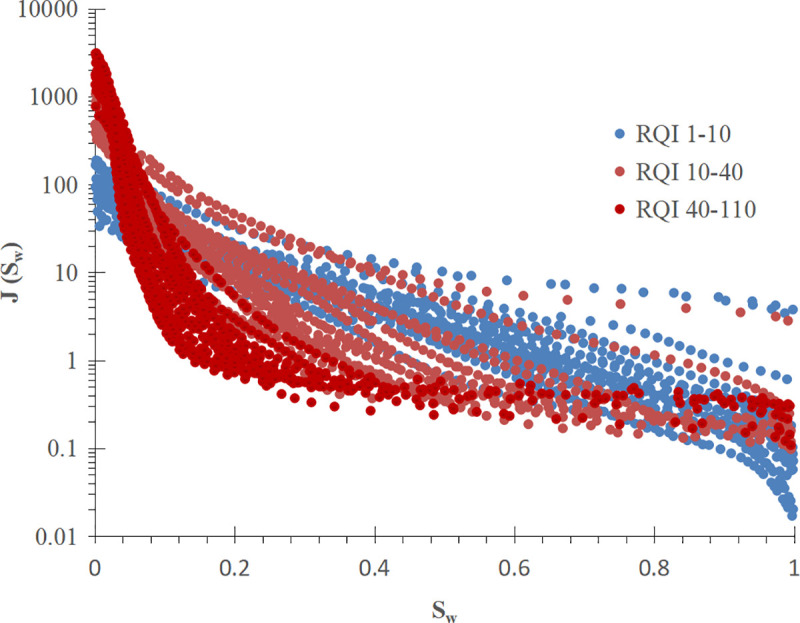
J-function curves of 55 cores from the N reservoir of the Zagros Basin.

**Fig 4:**
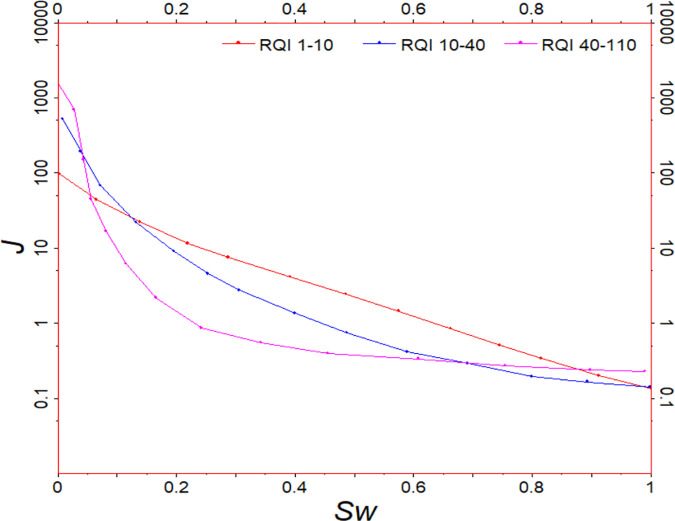
Pseudo-J-function curves of 55 cores from the N reservoir of the Zagros Basin.

In order to find the key parameters affecting the curve shape, firstly, the J-function curves of 55 cores in N reservoir of Zagros Basin are divided into three groups according to the distribution range of reservoir quality index (RQI) by using the idea of conventional J-function application, and each group of J-function curves is fitted (Fig. 3 and Fig. 4). From the shape of the pseudo-J-function curve, we can see that the larger the RQI is, the more the J-function curve tends to be a power function type; the smaller the RQI is, the more the J-function curve tends to be an exponential function type.

The porosity, permeability and RQI of the cores corresponding to the two types of J-function curves are shown in Table 1. It can be seen that the average porosity, average permeability and average RQI of the cores corresponding to the power function J-function curve are larger than those of the exponential function J-function curve. It can be firstly demonstrated that the factors affecting the shape of J-function curve are porosity, permeability and RQI.

**Table 1:** Physical parameters of cores corresponding to J-function curves.

J-function curve type	Porosity		Permeability(10^−3^μm^2^)		RQI(μm)	
	Range	Average	Range	Average	Range	Average
Power-function type	0.07 ~ 0.23	0.18	0.29 ~ 2604.54	657.65	2.02 ~ 108.92	47.84
Exponential-function type	0.05 ~ 0.19	0.13	0.08 ~ 29.73	4.69	1.34 ~ 17.83	4.83

**Fig 5:**
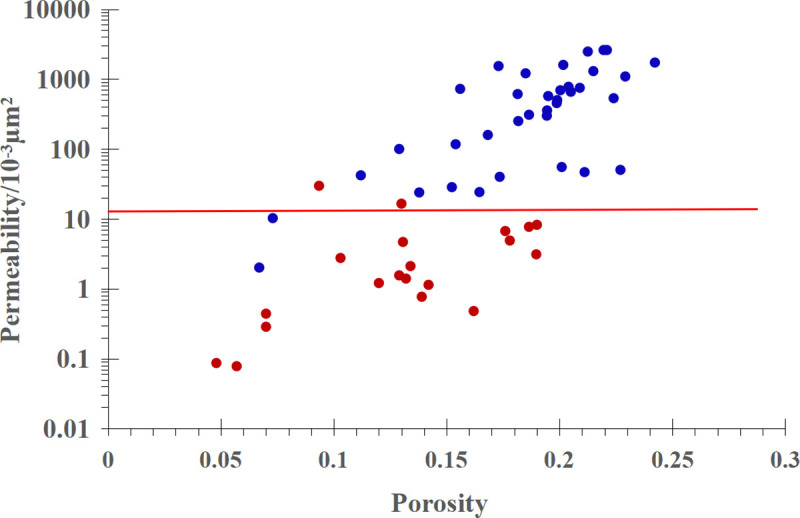
Cross plot of porosity and permeability of cores corresponding to two types of J-function curves.

The RQI of the cores corresponding to the above two types of J-function curves overlap with each other, so there is still great uncertainty in classifying the J-function curves only by RQI. Since RQI is a function of both porosity and permeability, consider using both porosity and permeability to classify the J-function curve. Fig 5 is a cross plot of porosity and permeability of cores corresponding to two types of J-function curves. The data points corresponding to the power function J-function curve are almost all above the red line, and the data points corresponding to the exponential function J-function curve are almost all below the red line. The red line corresponds to a permeability of 13.5 × 10^−3^μm^2^. Therefore, 13.5 × 10^−3^μm^2^ can be used as a criterion to classify the type of J-function curve.

The permeability of the target interval of a single well can be obtained by logging and other techniques, and then the J-function corresponding to each sampling point in the target interval can be obtained according to the definition of J-function. The J-function corresponding to the non-coring target interval of N reservoir in Zagros Basin can be classified by using 13.5 × 10^−3^μm^2^.

### 2.2. Improved J-function and water saturation calculation method

#### 2.2.1. Improved power function type J-function and water saturation calculation method.

**Fig 6:**
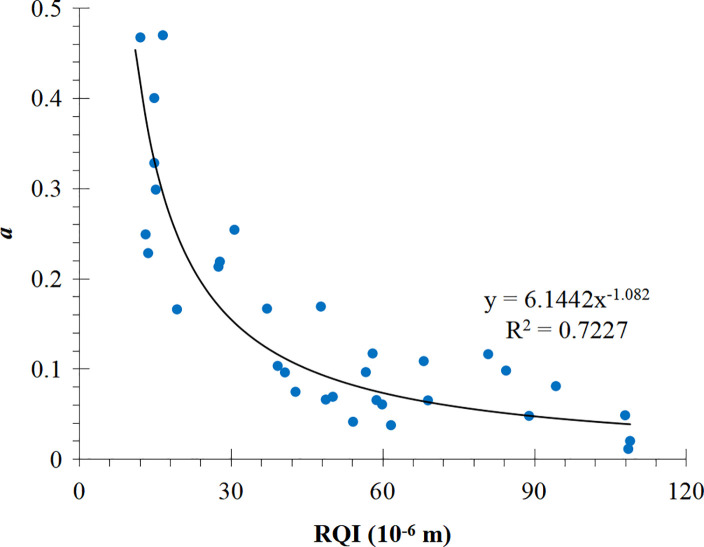
Relationship between a and RQI.

**Fig 7:**
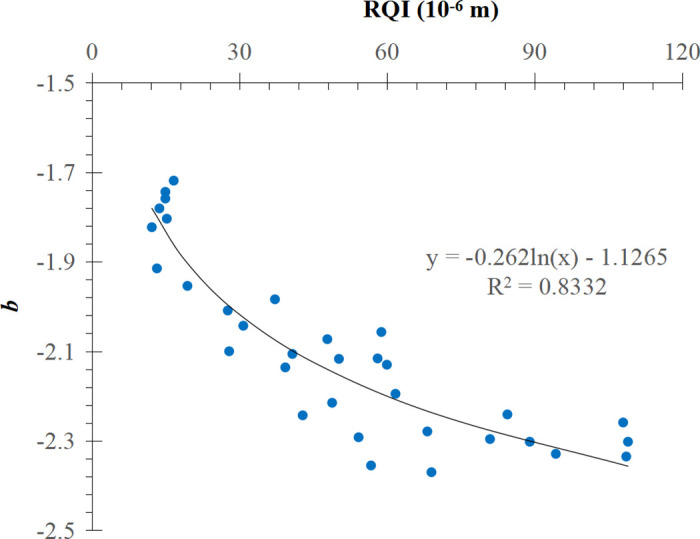
Relationship between b and RQI.


There are 35 J-function curves of power function types in total, and 2 of them are abnormal curves, so coefficients a and b are obtained by fitting the J-function curves of 33 cores after elimination. Both a and b were negatively correlated with RQI (Figs 6 and 7). When RQI is small (10  ~ 30 ), a and b change dramatically, when RQI is large (60 ~ 90), a and b change gently, when RQI is greater than 90, a and b almost do not change. For the J-function curve of power function type, when the reservoir physical properties are poor, the change of physical properties has a greater impact on the shape of J-function curve; when the reservoir physical properties are good, the change of physical properties has a smaller impact on the shape of J-function curve. Therefore, the traditional J-function curve grouping method often produces large errors in the reservoir intervals with poor physical properties. The functional relations between a, b and RQI are as follows:

$$a\;=\text{6}.\text{1442 }{\left(\sqrt{\frac{K}{\emptyset }}\right)}^{-1.082}$$ (13)

$$b=-\text{0}.\text{262ln}\sqrt{\frac{K}{\emptyset }}-1.1265$$ (14)

The mathematical model of the power function type J-function curve is obtained from the formulas (13 ),(14 ), and (3):


$$\text{J}=\left[\text{6}.\text{1442 }{\left(\sqrt{\frac{K}{\emptyset }}\right)}^{-1.082}\right]{S_{\text{w}}}^{-\text{0}.\text{262ln}\sqrt{\frac{K}{\emptyset }}-1.1265}$$ (15)

The initial water saturation model corresponding to the power function type J-function curve is obtained from Formula (10), Formula (13) and Formula (14):


$$S_{\text{w}}=S_{\text{wi}}+(1-S_{\text{wi}}){\left\{\frac{mn\Delta \rho gH}{\left[\text{6}.\text{1442 }{\left(\sqrt{\frac{K}{\emptyset }}\right)}^{-1.082}\right]\sigma_{\text{res}}\;\text{cos}\;\theta_{\text{res}}}\sqrt{\frac{K}{\emptyset }}\right\}}^{\frac{-1}{\text{0}.\text{262ln}\sqrt{\frac{K}{\emptyset }}+1.1265}}\text{\textbackslash{} \textbackslash{} }$$ (16)

Its further simplified form is as follows:


$$S_{\text{w}}=S_{\text{wi}}+(1-S_{\text{wi}}){\left[\frac{mn\Delta \rho gH{\left(\frac{K}{\emptyset }\right)}^{1.041}}{\text{6}.\text{1442}\sigma_{\text{res}}\;\text{cos}\;\theta_{\text{res}}}\right]}^{\frac{-1}{\text{0}.\text{262ln}\sqrt{\frac{K}{\emptyset }}+1.1265}}$$ (17)

**Fig 8:**
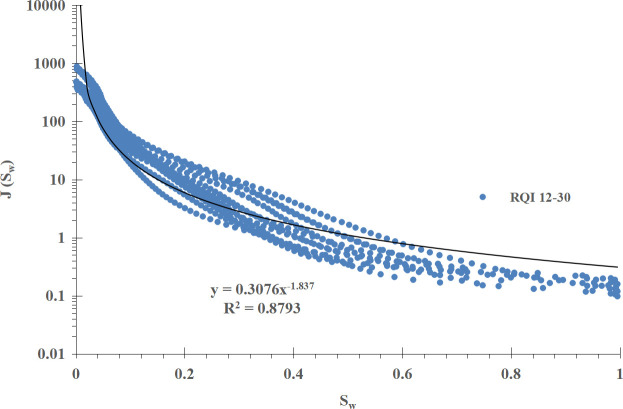
Group I J-function curve of power function type (RQI 12-30).

**Fig 9:**
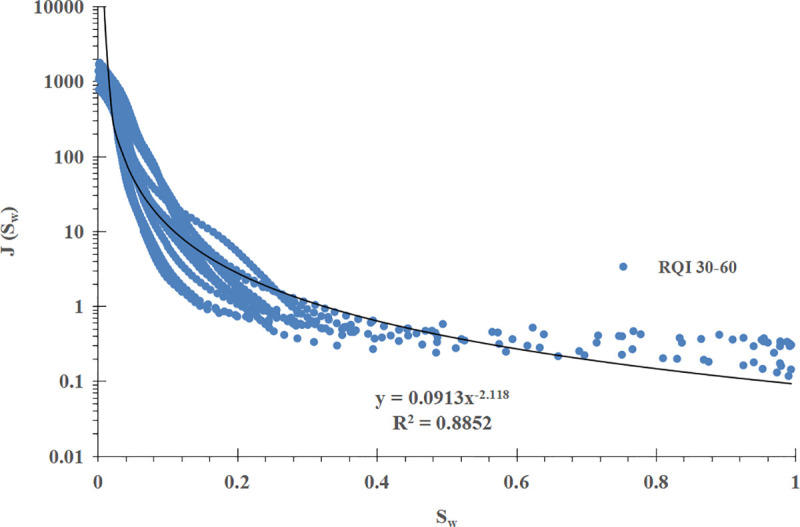
Group II J-function curve of power function type (RQI 30-60).

**Fig 10:**
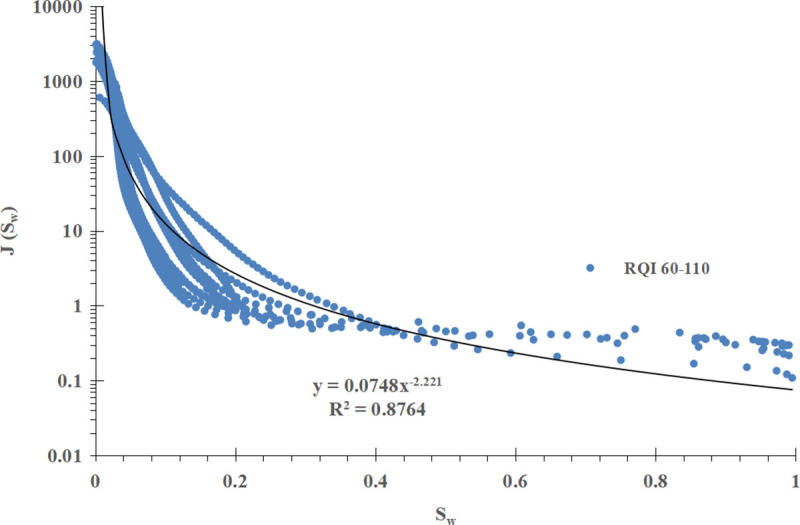
Group III J-function curve of power function type (RQI 60–110).

For comparison, the conventional method is used to divide the power function J-function curve into three groups according to the RQI range, and the mathematical fitting is carried out on them. The results are shown in Figs 8–10 respectively.μmμ

The mathematical models of the three sets of power function type J-function curves and the corresponding initial water saturation models are shown in Table 2.

**Table 2:** Mathematical model of power function type J-function curve and corresponding initial water saturation model.

Power function type J-function curve grouping	Mathematical model of J-function curves	Model of initial water saturation
I (RQI 12 ~ 30)	$\text{J}=\text{0}.\text{3076}{{\text{S}}_{\text{w}}}^{-\text{1}.\text{837}}$ (18)	$S_{\text{w}}={\left(\text{0}.\text{0117}\Delta H\sqrt{\frac{K}{\emptyset }}\right)}^{\frac{-\text{1}}{\text{1}.\text{837}}}$ (19)
II (RQI 30 ~ 60)	$\text{J}=\text{0}.\text{0913}{{\text{S}}_{\text{w}}}^{-\text{2}.\text{118}}\text{\textbackslash{} \textbackslash{} }$ (20)	$S_{\text{w}}={\left(\text{0}.\text{0395}\Delta H\sqrt{\frac{K}{\emptyset }}\right)}^{\frac{-\text{1}}{\text{2}.\text{118}}}$ (21)
III (RQI 60 ~ 110)	$\text{J}=\text{0}.\text{0748}{{\text{S}}_{\text{w}}}^{-\text{2}.\text{221}}\text{\textbackslash{} \textbackslash{} }$ (22)	$S_{\text{w}}={\left(\text{0}.\text{0482}\Delta H\sqrt{\frac{K}{\emptyset }}\right)}^{\frac{-\text{1}}{\text{2}.\text{221}}}$ (23)

Compared with the improved J-function method, the conventional J-function method divides the J-function curves into three groups, and a and b in the mathematical model and water saturation model of each J-function curve are constant and have the meaning of “average”, which is not enough to reflect the subtle changes of physical properties, especially for reservoirs with poor physical properties; The improved J-function method does not need to group the J-function curve, but establishes the mathematical model of a and b, which change with the change of RQI, from “discrete” data points to “continuous” data volume, which can “tailor” a and b corresponding to each geological unit in the reservoir, and can reflect the subtle changes of reservoir physical properties, which is more reasonable.

#### 2.2.2. Improved exponential function type J-function and water saturation calculation method.

**Fig 11:**
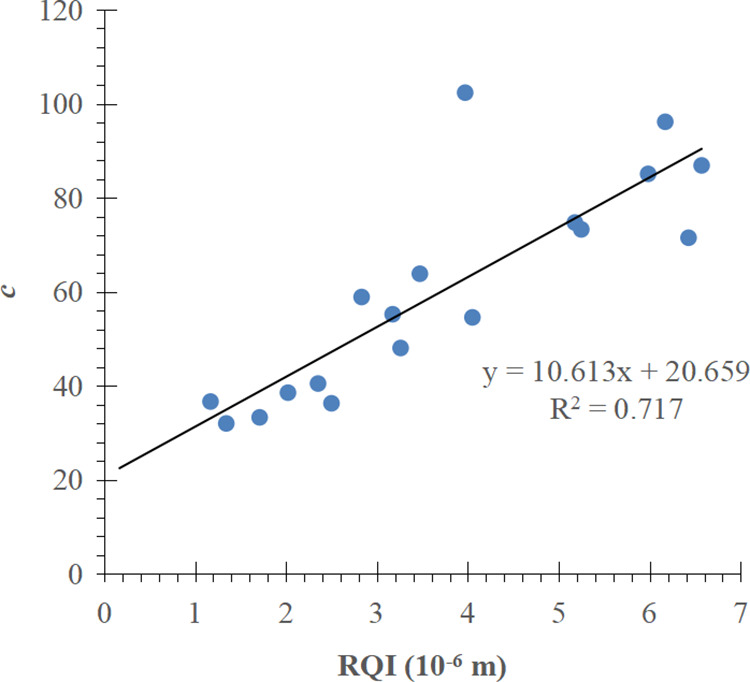
Relationship between c and RQI.

**Fig 12:**
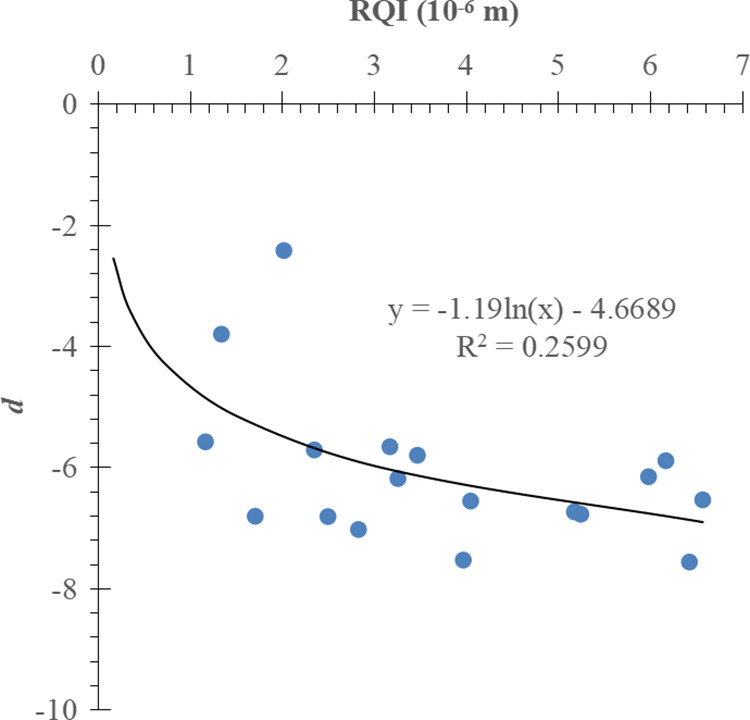
Relationship between d and RQI.


There are 20 J-function curves of exponential function type in total, and 2 of them are abnormal curves, so the remained J-function curves of 18 cores are fitted after the 2 abnormal curves are eliminated, and c and d are obtained. The goodness of fit of d needs to be improved due to the lack of J-function curves of exponential function type. The analysis showed a positive correlation between c and RQI and a negative correlation between d and RQI (Figs 11 and 12). The functional relations between c, d and RQI are as follows:

$$c=\text{10}.\text{613}\sqrt{\frac{K}{\emptyset }}\;+\text{ 20}.\text{659}$$ (24)

$$d=-\text{1}.\text{19ln}\left(\sqrt{\frac{K}{\emptyset }}\right)-4.6689$$ (25)

The mathematical model of exponential function type J-function curve is obtained from the equations (11), (24), and (25):


$$\text{J}=\left(\text{10}.\text{613}\sqrt{\frac{K}{\emptyset }}+\text{ 20}.\text{659\textbackslash{} }\right)e^{\left[-\text{1}.\text{19ln}(\sqrt{\frac{K}{\emptyset }})\;-\text{ 4}.\text{6689}\right]S_{w}}$$ (26)

The water saturation model corresponding to the J-function curve of exponential function type is obtained from Formula (12), Formula (24) and Formula (25):


$$S_{\text{w}}=S_{\text{wi}}+(1-S_{\text{wi}})\frac{\text{ln}(\frac{mn\Delta \rho g\Delta H\;}{\left(\text{10}.\text{613}\sqrt{\frac{K}{\emptyset }}+\text{ 20}.\text{659\textbackslash{} }\right)\sigma_{\text{res}}\;\text{cos}\;\theta_{\text{res}}\;}\sqrt{\frac{K}{\emptyset }})}{-\text{1}.\text{19ln}(\sqrt{\frac{K}{\emptyset }})\;-\text{ 4}.\text{6689}}$$ (27)

**Fig 13:**
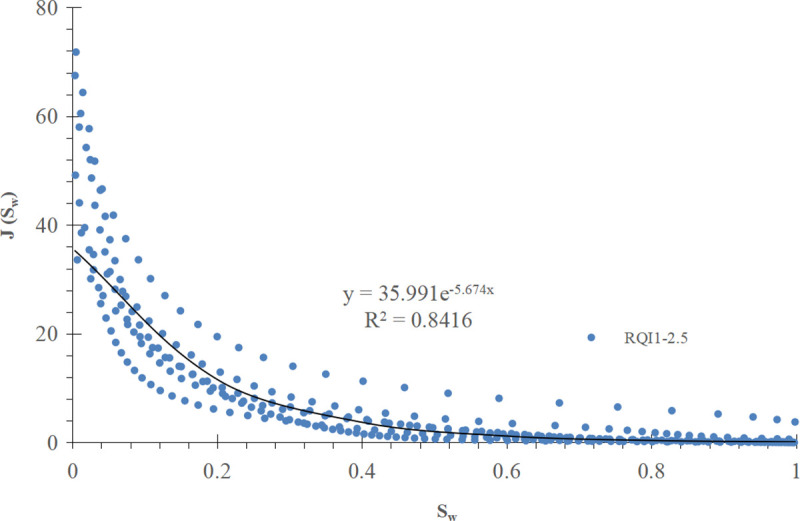
Group I J-function curve of exponential function type (RQI 1-2.5).

**Fig 14:**
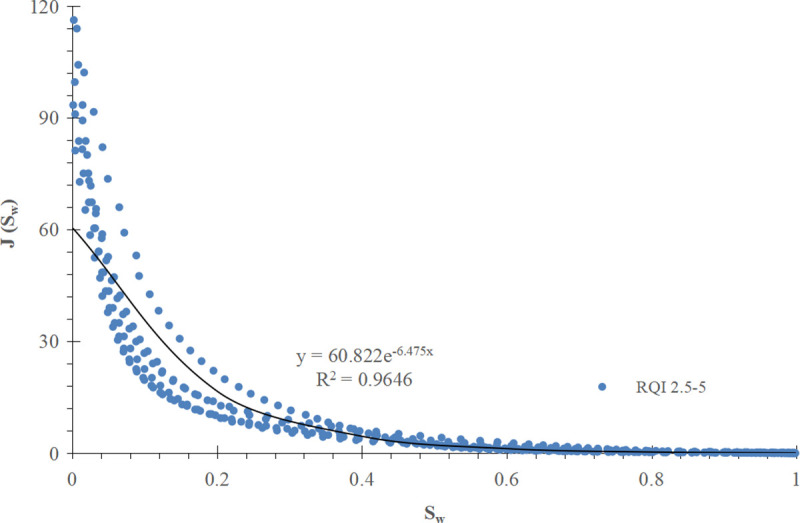
Group II J-function curve of exponential function type (RQI 2.5-5).

**Fig 15:**
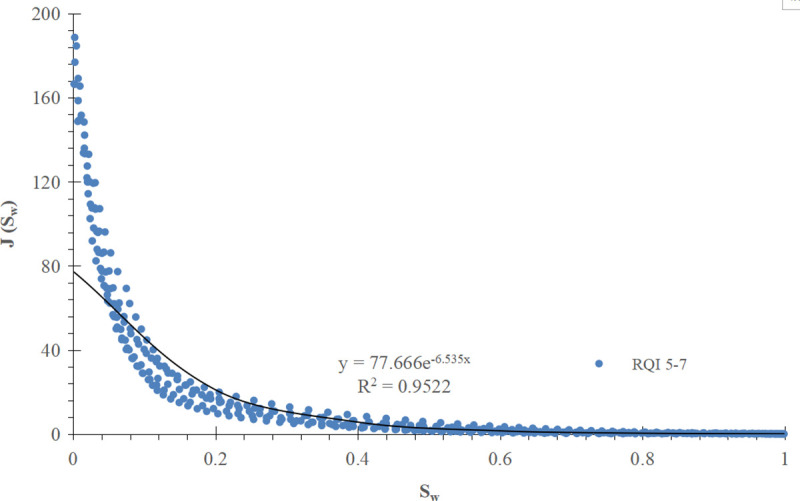
Group III J-function curve of exponential function type (RQI 5-7).

For comparison, the conventional method is used to divide the J-function curve of exponential function type into three groups according to the RQI range, and the mathematical fitting is carried out, as shown in Figs 13–15 respectively.

Mathematical models and corresponding water saturation models of three groups of exponential function type J-function curves are shown in Table 3 below:

**Table 3:** Mathematical model of exponential function type J-function curve and corresponding initial water saturation model.

Grouping of J-function curves of exponential function type	Mathematical model of J-function curves	Model of initial water saturation
I(RQI 1-2.5)	$\text{J}=\text{35}.\text{991}e^{-\text{5}.\text{674}S_{w}}$ (28)	$\begin{array}{c} S_{\text{w}}=\frac{\text{ln}\left(10^{-4}\Delta H\sqrt{\frac{K}{\emptyset }}\right)}{-5.674} \end{array}$ (29)
II(RQI 2.5-5)	$\text{J}=\text{ 60}.\text{822}e^{-\text{6}.\text{475}S_{w}}$ (30)	$\begin{array}{c} S_{\text{w}}=\frac{\text{ln}\left(5.92\times 10^{-5}\Delta H\sqrt{\frac{K}{\emptyset }}\right)}{-6.475} \end{array}$ (31)
III(RQI 5–7)	$\text{J}=\text{77}.\text{6661}e^{-\text{6}.\text{535}S_{w}}$ (32)	$\begin{array}{c} S_{\text{w}}=\frac{\text{ln}\left(4.64{\times 10}^{-5}\Delta H\sqrt{\frac{K}{\emptyset }}\right)}{-6.535} \end{array}$ (33)

The results show that the improved exponential function type J-function has the same optimization effect as the power function type J-function. For the corresponding reservoir, c and d in the water saturation model established by the conventional J-function method are constant values, which have the meaning of “average” and can not reflect the subtle changes of physical properties; The c and d in the initial water saturation model established by the improved J-function method change with the change of reservoir physical properties, from “discrete” data points to “continuous” data volume, which has the meaning of “tailor-made” and can reflect the subtle changes of reservoir physical properties.

## 3. Case study

**Fig 16:**
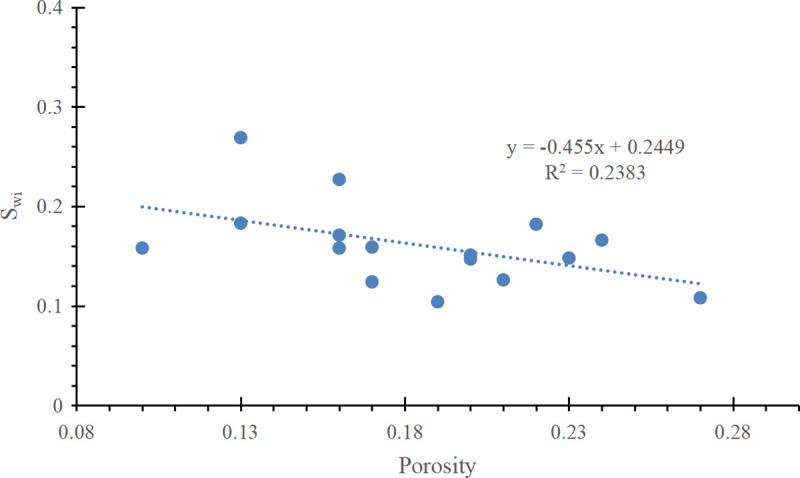
The relationship between Porosity and S_wi._


Reservoir N in Zagros Basin, Iraq is a sandstone reservoir, with porosity ranging from 0.05 to 0.24 and an average of 0.16, and permeability ranging from 0.08 × 10^−3^μm^2^ to 8000 × 10^−3^μm^2^ and an average of 436 × 10^−3^μm^2^. In this study, the field unit system is used, where, *m* = 0.2166, *n* = 1.4214, Δ*ρ* = 0.3043g/cm^3^, $\sigma_{\text{res}}\;\text{cos}\;\theta_{\text{res}}$ = 26dyn/cm. S_wi_ of another 16 core samples were tested by Corelab. S_wi_ is a function of the porosity, as shown in Formula (34) and Fig 16.

$${\text{S}}_{\text{wi}}=-\text{0}.\text{455}\emptyset +\text{0}.\text{2449}$$ (34)

Substitute the above parameters into Formula (17), Formula (27) and Formula (34) to obtain the improved initial water saturation model corresponding to the power function type and exponential function type J-function curves, as shown in Formula (35) and Formula (36) respectively:


$$S_{\text{w}}=-\text{0}.\text{455}\emptyset +\text{0}.\text{2449}+({\text{0}.\text{455}\emptyset +\text{0}.\text{7551})\left[5.86{\;*\;10}^{-4}\Delta H{\left(\frac{K}{\emptyset }\right)}^{1.041}\right]}^{\frac{-1}{0.262\text{ln}\sqrt{\frac{K}{\emptyset }}+1.1265}}$$ (35)

$$S_{\text{w}}=-\text{0}.\text{455}\emptyset +\text{0}.\text{2449}+(\text{0}.\text{455}\emptyset +\text{0}.\text{7551})\frac{\text{ln}(\frac{0.0036\Delta H\;\sqrt{\frac{K}{\emptyset }}}{\text{10}.\text{613}\sqrt{\frac{K}{\emptyset }}\;+\text{ 20}.\text{659}})}{-\text{1}.\text{19ln}\sqrt{\frac{K}{\emptyset }}\;-\text{ 4}.\text{6689}}$$ (36)

**Fig 17:**
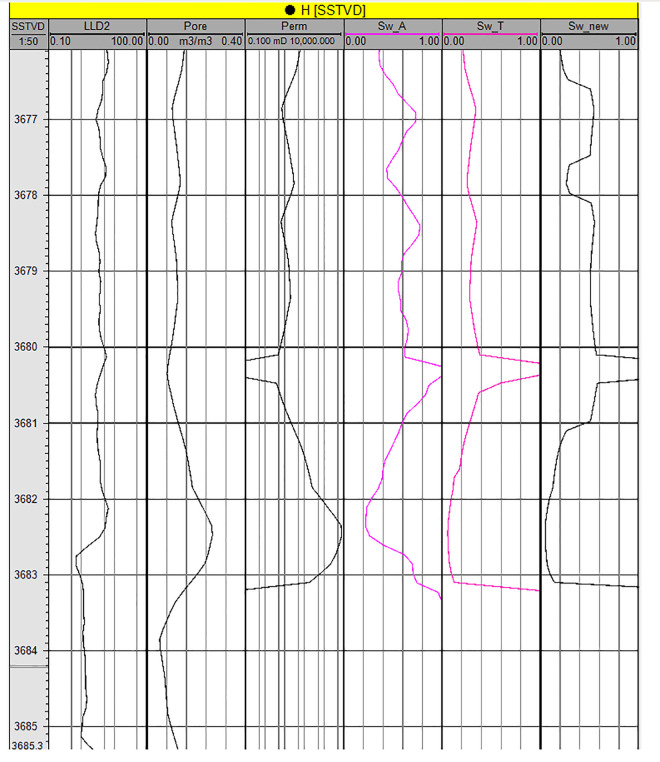
Water saturation curve of each interval of Well H in N reservoir under different interpretation methods.

The above initial water saturation model is applied in N reservoir of Zagros Basin in Iraq, and H well is taken as an example. Fig 17 shows the water saturation curve of Well H in N reservoir of Zagros Basin, from left to right, it is resistivity, porosity, permeability, water saturation of Archie method, water saturation of conventional J-function method and water saturation of improved J-function method. Table 4 shows the water saturation calculated by different methods for different intervals of Well H. According to Fig 17 and Table 4, the three interpretation methods show very similar results in intervals with medium physical properties (porosity 0.13-0.18 and permeability 20 × 10 ⁻ ^3^μm^2^-200 × 10 ⁻ ^3^μm^2^). In poorer-quality intervals (3676.5-3677.5 m, 3678–3680 m, and 3680.5-3681 m), both the Archie method and improved J-function method yield similar water saturation values, while the conventional J-function method gives relatively lower saturation values. The conventional method adopts the “average value” of a, b, c and d, which can not reflect the slight change of physical properties. In the interval with relatively good physical properties (3682–3683 m), the water saturation interpreted by the conventional J-function method and the modified J-function method is generally consistent and significantly lower than that derived from the Archie equation. This interval is located 130 m above the oil-water contact, far from the transition zone, where the water saturation is nearly equal to the irreducible water saturation. Core displacement experiments indicate that the irreducible water saturation for such high-porosity, high-permeability reservoirs is approximately 0.1 (Fig 16). The modified J-function method yields a water saturation of 0.06–0.11, while the conventional J-function method gives 0.05–0.13, both closely matching the experimental value of 0.1. In contrast, the Archie equation results in a water saturation of 0.23–0.74, which is significantly higher than 0.1 and contradicts the displacement experiments. Therefore, the improved S_w_ calculation accuracy will significantly impact completion design and production forecasting.

**Table 4:** Water saturation of each interval of H well in N reservoir under different interpretation methods.

Depth(m)	Porosity	Permeability(10^−3^μm^2^)	Archie’s method for water saturation	Conventional J-function method for water saturation	Improved J-function method for water saturation
3676.5 ~ 3677.5	0.10 ~ 0.12	8.0 ~ 18.0	0.55 ~ 0.73	0.29 ~ 0.34	0.51 ~ 0.54
3678.0 ~ 3680.0	0.10 ~ 0.13	6.5 ~ 19.7	0.60 ~ 0.77	0.30 ~ 0.36	0.52 ~ 0.55
3680.5 ~ 3681.0	0.09 ~ 0.13	3.8 ~ 19.2	0.59 ~ 0.83	0.29 ~ 0.37	0.51 ~ 0.56
3682.0 ~ 3683.0	0.18 ~ 0.27	200 ~ 8000	0.23 ~ 0.74	0.05 ~ 0.13	0.06 ~ 0.11

In summary, for Reservoir N, within the porosity range of 0.1–0.27, the modified J-function method improves the accuracy of original water saturation interpretation compared to the Archie equation and the traditional J-function method. However, despite its theoretical advancements over the conventional J-function model, the modified J-function method still has three limitations: First, limited experimental data, leading to underestimated fitting coefficients (a, b, c, d); Second, no closed coring has been conducted in Reservoir N, meaning there is no definitive experimental data to validate the accuracy of the modified water saturation model; Third, the findings are currently applicable only to Reservoir N and have not been verified in other reservoirs. The methodology presented in this study may serve as a reference for peers in the field.

## 4. Conclusion

(1) By studying 55 J-function curves of the N oil reservoir in the Zagros Basin, it is determined that permeability is the main controlling factor affecting the shape of the J-function curve. Based on this, a classification method and criteria for J-function curves are further established, according to which J-function curves can be divided into power function type and exponential function type.
(2) The undetermined coefficients of the improved power function type and exponential function type J-function have a functional relationship with RQI, forming a “continuous” data volume that can reflect the slight changes in reservoir physical properties, achieving a tailored approach for any reservoir.
(3) The improved J-function and the improved initial water saturation model do not require grouping, overcoming the shortcomings of the traditional J-function method. Even in relatively poor physical property reservoirs, they still maintain good calculation accuracy.
(4) To advance this research, future work should focus on expanding the core dataset to improve the statistical reliability of coefficients a-d, while also incorporating pressurized core analysis in the N Reservoir to rigorously validate the improved model’s accuracy. Furthermore, the methodology’s generalizability should be assessed by testing its performance across diverse reservoir types with varying petrophysical characteristics, including both carbonate and clastic formations, to develop robust universal correction factors that extend beyond the current N Reservoir application.

## Supporting information

S1 File Inclusivity-in-global-research-questionnaire. (DOCX)

S2 File Data set used to conduct this study and generate this manuscript. (XLSX)
